# Ingestion accidentelle d'une pièce de monnaie en intra-oesophagien

**DOI:** 10.11604/pamj.2015.21.324.7712

**Published:** 2015-08-31

**Authors:** Rachid Marouf

**Affiliations:** 1 Service de Chirurgie Thoracique, CHU Mohammed VI, Oujda, Maroc

**Keywords:** Corps étranger, pièce de monnaie, bronchoscopie rigide, Foreign body, coin, rigid bronchoscopy

## Image en medicine

Il s'agit d'une jeune fille de 3 ans, sans antécédent particulier, admise aux urgences du CHU Mohammed VI d'Oujda dans un tableau de dysphagie aux solides avec hyper-sialorrhée, l'interrogatoire a trouve la notion d'ingestion accidentelle d'une énorme pièce de monnaie enclavée à l'entrée de l'oesophage. La réalisation d'une radiographie thoracique de face a montré la présence d'un énorme objet radio-opaque se projetant au niveau de la région cervicale, le cliché de profil confirmé la position postérieure du CE œsophagien par rapport aux clartés antérieures du larynx, de la trachée et de la carène. L'extraction a été faite au bloc opératoire par l'utilisation d'une endoscopie oesophagienne. Les suites ont été simples. Les ingestions de corps étrangers (CE) surviennent, Le plus souvent d'une façon accidentelle, dans la majorité des cas avant l’âge de 5 ans. Les symptômes liés à l'ingestion d'un CE dépendent de l’âge de l'enfant, de ses antécédents (chirurgie digestive), de la taille et de la localisation du CE et/ou de la survenue d'une complication éventuelle (ulcération, perforation digestive…). Si la plupart des CE ingérés traversent le tractus digestif sans manifestation clinique ni complication, 10 à 20% d'entre eux doivent être extraits par voie endoscopique en urgence et moins de 1% nécessite un traitement chirurgical en raison d'une complication majeure: soit une obstruction oesophagienne, ou une perforation pouvant être responsable d'une médiastinite.

**Figure 1:**
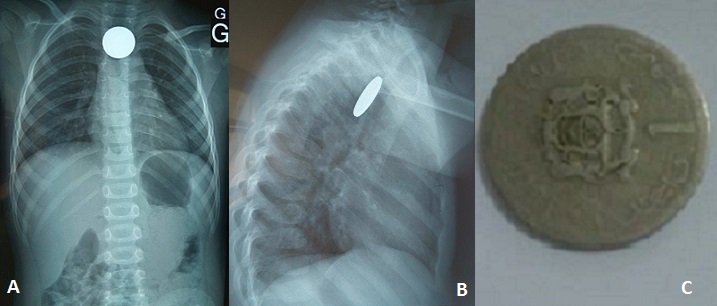
(A) radiographie thoracique de face qui montre un énorme objet radio-opaque se projetant au niveau de la région cervicale; (B) cliché de profil qui confirme la position postérieure du CE œsophagien par rapport aux clartés antérieures du larynx, de la trachée et de la carène; (C) la pièce de monnaie après extraction sous bronchoscopie rigide

